# Biomechanical Evaluation of the Transcortical and Transpedicular Trajectories for Pedicle Screw Insertion in Thoracolumbar Fracture Fixation for Ankylosing Spondylitis

**DOI:** 10.3389/fsurg.2021.706597

**Published:** 2021-09-08

**Authors:** Zhizhong Tong, Bin Xiao, Kai Yan, Yonggang Xing, Yanbin Zhang

**Affiliations:** ^1^Department of Radiology, Beijing Jishuitan Hospital, Beijing, China; ^2^Department of Spine Surgery, Beijing Jishuitan Hospital, Beijing, China; ^3^Department of Education, Beijing Jishuitan Hospital, Beijing, China

**Keywords:** ankylosing spondylitis, thoracolumbar fracture, transpedicular screw, cortical bone trajectory, biomechanical characteristics, finite element analysis

## Abstract

**Background:** Ankylosing spondylitis (AS) is a chronic disorder characterized by an imbalance between bone formation and resorption. Spinal fractures often occur after minor trauma in patients with AS. For thoracolumbar fractures, transpedicular screw (TPS) fixation through the posterior approach has been suggested. The cortical bone trajectory (CBT) technique has also been used to prevent screw pull-out in patients with poor bone quality. The aim of current study was to assess the biomechanical characteristics of the TPS and CBT technique in thoracolumbar AS fracture fixation by finite element analysis.

**Methods:** The three-dimensional finite element models of the AS spine were created. The CBT and TPS methods of screw insertion were used in AS spinal fracture models. An intact AS spine model was considered the control. An axial force and torsion in rotation, flexion/extension and lateral flexion were applied in all models in CBT, TPS, and control groups.

**Results:** The AS spine showed similar construct stiffness after posterior fixation by CBT and TPS techniques under axial, rotational, and flexion/extension loading conditions. The TPS technique showed better intact stability under all loading conditions. Similarly, the TPS technique provided superior fracture regional stability against axial and rotational loads than did the CBT technique. The maximum von Mises stresses were 1714.4 ± 129.8 MPa and 1208.7 ± 107.3 MPa (*p* < 0.001), which occurred in the CBT and TPS groups under compressive loading.

**Conclusions:** The TPS technique provides better biomechanical strength under axial, rotational, flexion/extension, and lateral flexion loading than does the CBT technique. Compared with CBT, TPS is more effective in maintaining the stability of AS thoracolumbar fractures from a finite element analysis perspective.

## Introduction

Ankylosing spondylitis (AS) is a chronic disorder characterized by immune-mediated inflammatory arthritis that mainly involves the axial skeleton and sacroiliac joints (1, 2). The obvious feature is an imbalance between bone formation and bone resorption in the skeletal system (3). As the disease progresses, the formation of bridging syndesmophytes between vertebrae eventually leads to stiff spines (4, 5). As a result, AS patients typically exhibit persistent backache and a stooped posture and have an unsatisfactory quality of life (5). Moreover, spinal fractures often occur after minor trauma (2, 6).

Spinal fractures most commonly occur in the cervical spine and thoracolumbar junction (6, 7). The stability of fractures, neurologic status and bone mineral density should be considered in the treatment of AS spinal fractures (7, 8). Conservative treatments, such as the cervical collar, halo vest, and thoracolumbrosacral orthosis, are not ideal for unstable spinal fractures (6, 8). The surgical indications usually include neurologic function deficits, unstable fracture fragments, and epidural hematoma (6–8). For thoracolumbar spinal fractures, transpedicular screw fixation through the posterior approach has been suggested (7, 8). To avoid adjacent vertebral fractures, the levels at least three levels above and below the fractural vertebra should be fixed with long structures (8, 9). However, pedicle screw fixation failure is still one of the most common complications that occur due to poor bone quality in AS patients (7, 10).

To improve the holding force of pedicle screws in the cancellous bone pathway and specifically prevent screw pull-out in osteoporotic bone, the cortical bone trajectory (CBT) technique was developed (11). The CBT pathway follows the medial-to-lateral direction and a caudocephalad trajectory in the middle and posterior parts of the vertebrae (11, 12). This technique leads to better contact at the bone-screw interface than does the traditional transpedicular approach. Several studies have demonstrated that the CBT technique can provide high pullout strength (13, 14), superior resistance to craniocaudal cyclic loading (15), and better biomechanical stability (16). Clinical studies have shown that excellent vertebral fusion and low screw failure rates have been achieved by the CBT technique (17–19). Theoretically, the CBT technique is a promising treatment for AS spinal fractures, with better biomechanical stability of the fracture fragments and a lower risk of screw fixation failure. However, the biomechanical outcomes of the new technique in thoracolumbar AS fracture fixation have not been investigated. Thus, the aim of the present study was to assess the biomechanical characteristics of CBT and the traditional transpedicular screw (TPS) technique for pedicle screw insertion in thoracolumbar AS fracture fixation by finite element analysis.

## Materials and Methods

### Finite Element Models

**Figure 1:**
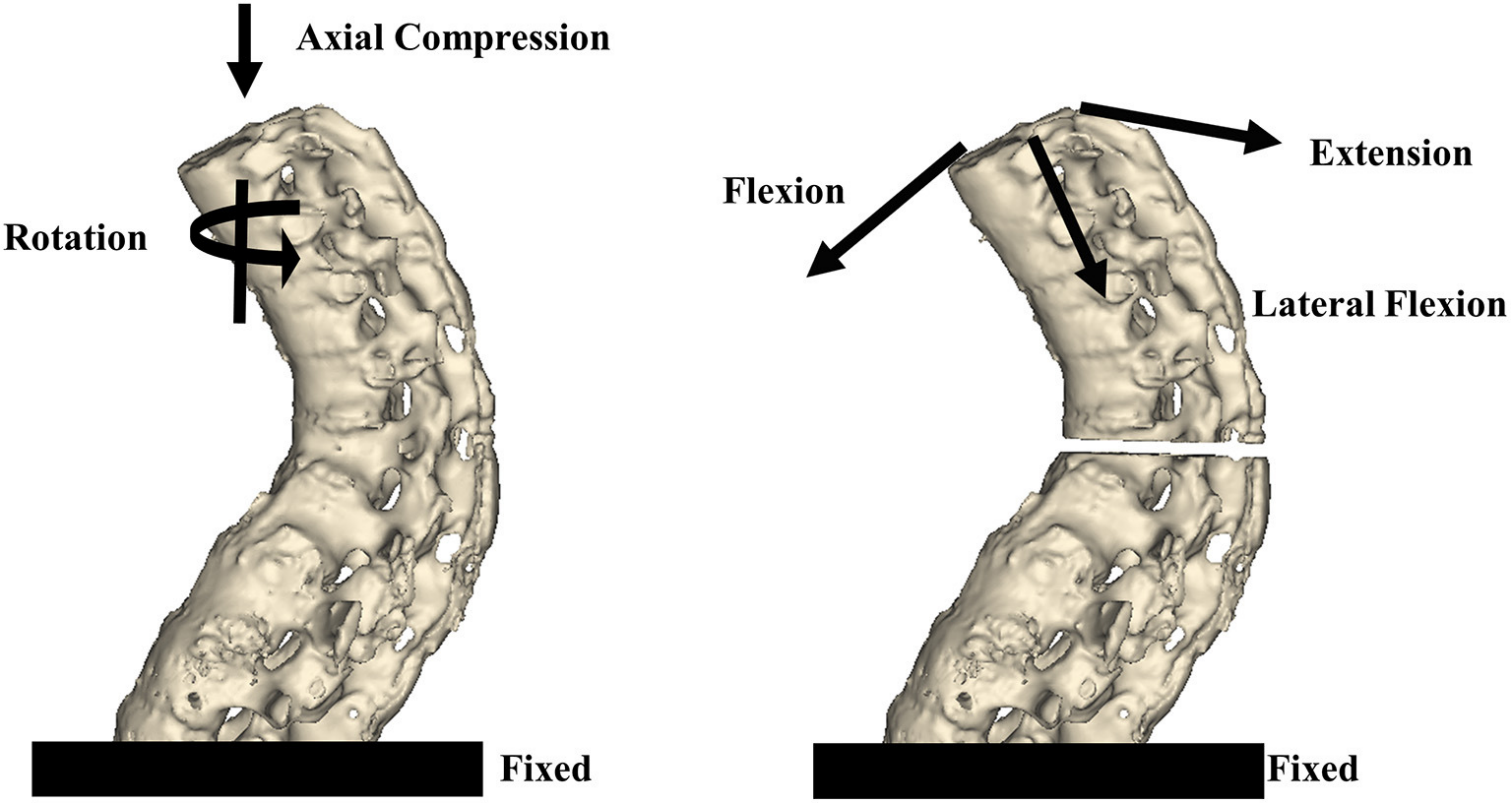
The thoracolumbar AS spine model from T8 to L4 was created on the basis of a CT scan. To simulate an unstable three-column thoracolumbar fracture, a horizontal osteotomy with a 5 mm bone defect at T12 was performed. An axial force and torsion were applied to all models. For the axial force, 300 N was applied to T8 and vertically downward in the coronal and sagittal planes. For torsion, a 10-Nm torque was applied to T8 around the vertical axis. To simulate flexion/extension and lateral flexion, a 10-Nm torque was applied to T8 toward the front/rear and side orientations.

The three-dimensional finite element models were created on the basis of the computed tomography (Lightspeed VCT, GE, Fairfield, CT) scans of AS volunteers. The thoracolumbar AS spine model from T8 to L4 was created from 10 volunteers with fusion spine and osteoporosis. An extreme unstable three-column thoracolumbar fracture was simulated (Figure 1). The vertebrae from T8 to L4 were complete fused. A horizontal osteotomy with a 5 mm bone defect at T12 vertebra was performed. The ligaments at fracture segments were completely destroyed.

Two methods of screw insertion were used in the AS spinal fracture model including the CBT and TPS techniques. The CBT technique was performed according to the methods reported in previous studies (11, 13, 16). Pedicle screws with lengths and diameters of 30–45 mm and 6.5 mm were selected according to the patients' individual characteristics. Twelve pedicle screws were inserted at the T9–L3 levels. The ipsilateral pedicle screws were connected by two longitudinal rods. The screws were not threaded to simplify the implant models. The properties of the cortical bone, cancellous bone, and implants are shown in Table 1. The AS spinal fracture model had a total of 952,964 elements and 249,366 nodes.

**Table 1:** Material properties of finite element models.

**Material**	**Elastic modulus (MPa)**	**Poisson's ratio**
**Bone**		
Cortical bone	12,000	0.3
Cancellous bone	500	0.3
Intervertebral osteophyte	12,000	0.3
Ligament	12,000	0.3
Implant	114,000	0.3

### Finite Element Analysis

The finite element analysis was performed by Abaqus 6.13 (Simulia, Providence, RI). Linear elastic isotropic material properties were assigned to all models and implants. The pedicle screws were locked to the bone. The implant interfaces were set to have rigid bonds. All contact elements were defined as deformable elements. The finite element analysis was performed under frictionless conditions in a simplified analysis model.

The intact AS spine models were included in the control group (*n* = 10). The fractured spine was fixed by screws through the traditional transpedicular pathway in the TPS group (*n* = 10). The CBT technique was applied to fix the fractures in the CBT group (*n* = 10). The L4 was fixed in the models. Axial forces and torsion were applied to all models in the three groups. For the axial force, 300 N loading was applied to T8 and vertically downward in the coronal and sagittal planes. For torsion, a 10-Nm torque was applied to T8 around the vertical axis. To simulate flexion/extension and lateral flexion, a 10-Nm torque was applied to T8 toward the front/rear and side orientations.

The biomechanical characteristics of the thoracolumbar AS spine in the immediate post-operative period were assessed in the present study. Axial and rotational construct stiffness was assessed to determine the integral stability of the thoracolumbar spine. The regional stability of the fractural segments was evaluated on the basis of the relative displacement and rotational angle between the proximal and distal segments. The effects of stress on the spine and implants were assessed by the von Mises stress distribution and maximum stresses.

### Statistical Analysis

Statistical analyses were performed with SPSS (version 19.0; SPSS Inc., Chicago, IL) software. One-way analysis of variance (ANOVA) was used to compare the means among the three groups. The independent-samples *t*-test was used to compare the means between the CBT and TPS groups. The level of statistical significance was defined as *p* < 0.05.

## Results

### The Spine Construct Stiffness

**Figure 2:**
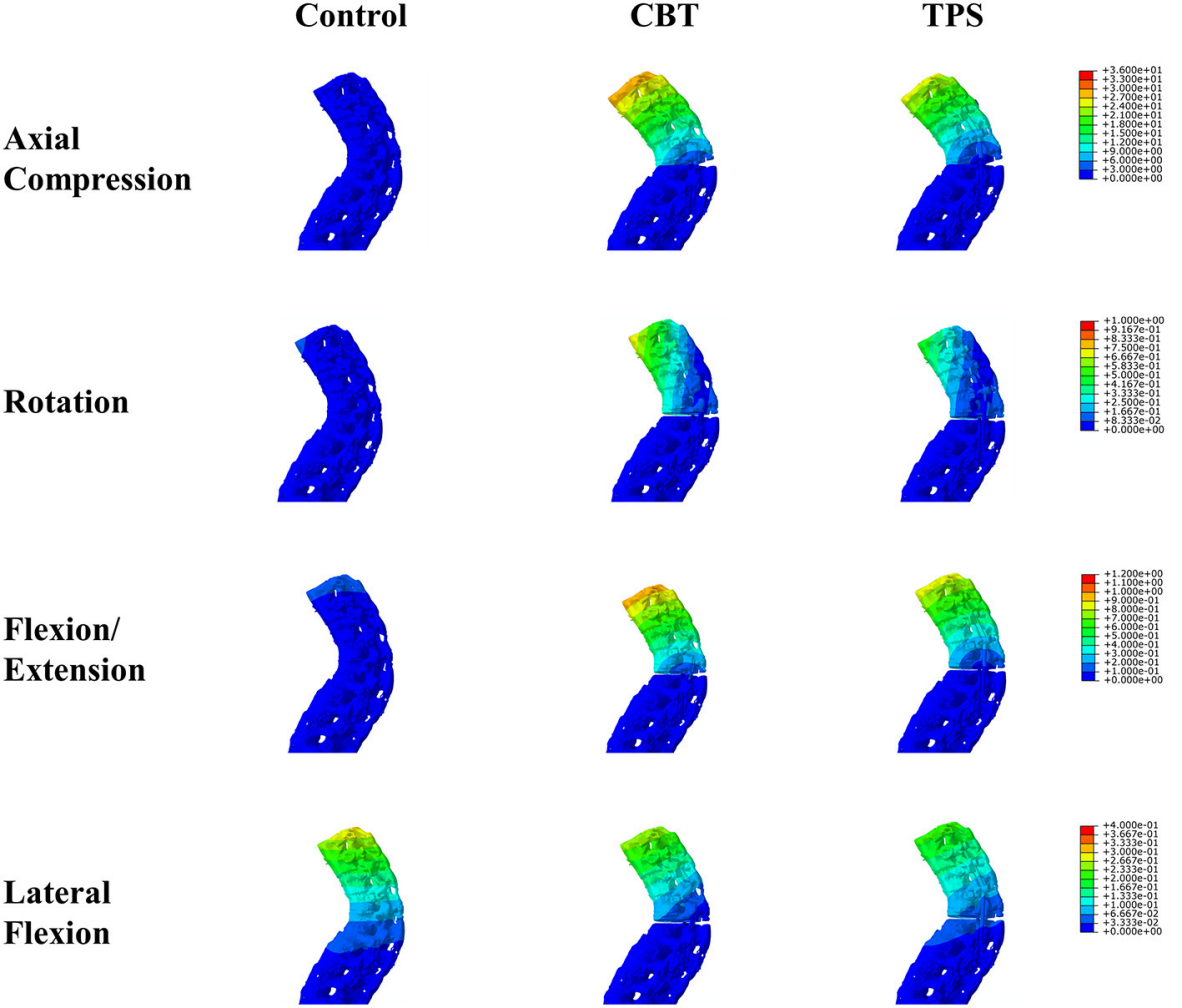
Spinal displacements under axial compression, rotation, flexion/extension, and lateral flexion loads.

The construct stiffnesses under different load conditions are shown in Table 2 and Figure 2. The AS spine showed similar construct stiffness after posterior fixation by CBT and TPS techniques under axial, rotational, and flexion/extension loading conditions. The traditional TPS technique showed better construct stiffness; however, a significant difference between groups occurred only in the rotational loading condition (21.0 ± 0.8 and 15.6 ± 0.7 N/mm, *p* < 0.001). Under the lateral flexion loading condition, the fixed spine group exhibited higher construct stiffness than did the control group (*p* < 0.001), while the traditional transpedicular technique yielded higher construct stiffness than did the CBT technique (*p* < 0.001). The TPS technique showed better intact stability under the loading conditions in the present complete fusion spine models.

**Table 2:** The construct stiffnesses under different load conditions.

**Load condition**	**Control**	**CBT**	**TPS**	p*-value
Axial compression (N/mm)	118.6 ± 19.6	11.7 ± 1.6	12.5 ± 1.2	<0.001
Rotation (Nm/deg)	72.9 ± 3.4	15.6 ± 0.7	21.0 ± 0.8	<0.001
Flexion/extension (Nm/deg)	119.1 ± 12.1	17.7 ± 1.7	19.0 ± 1.8	<0.001
Lateral flexion (Nm/deg)	48.9 ± 2.1	81.7 ± 2.5	109.6 ± 4.8	<0.001

*CBT, Cortical Bone Trajectory; TPS, Transpedicular Screw*.

### Fracture Regional Stability

The results of regional stiffness in the fracture region are shown in Table 3. The local stiffness in the TPS group was higher than the CBT group under rotation and lateral flexion condition (*p* < 0.001). The magnitude of micromotion between the proximal and distal fracture fragments under axial loads was 3.1 ± 1.3 mm in the TPS group and 3.8 ± 0.9 mm in the CBT group, and the difference was not significant (*p* = 0.193). Under the rotational and lateral flexion loading conditions, a higher relative rotation angle between the fracture fragments was shown in the TPS group (*p* < 0.001). Similar regional stability was observed in the CBT and TPS groups under flexion/extension loads (0.340 ± 0.017 and 0.358 ± 0.022 degrees, *p* = 0.066). The TPS technique provided superior stability against axial and rotational loads than did the CBT technique in the present complete fusion spine models.

**Table 3:** The local stability under different load conditions.

**Load condition**	**CBT**	**TPS**	***p-*value**
Axial compression (N/mm)	32.3 ± 3.7	37.3 ± 4.2	0.089
Rotation (Nm/deg)	28.3 ± 1.0	40.3 ± 1.4	<0.001
Flexion/extension (Nm/deg)	28.1 ± 1.7	29.4 ± 1.5	0.069
Lateral flexion (Nm/deg)	161.8 ± 33.4	467.2 ± 112.9	<0.001

*CBT, Cortical Bone Trajectory; TPS, Transpedicular Screw*.

### The Von Mises Stress

**Figure 3:**
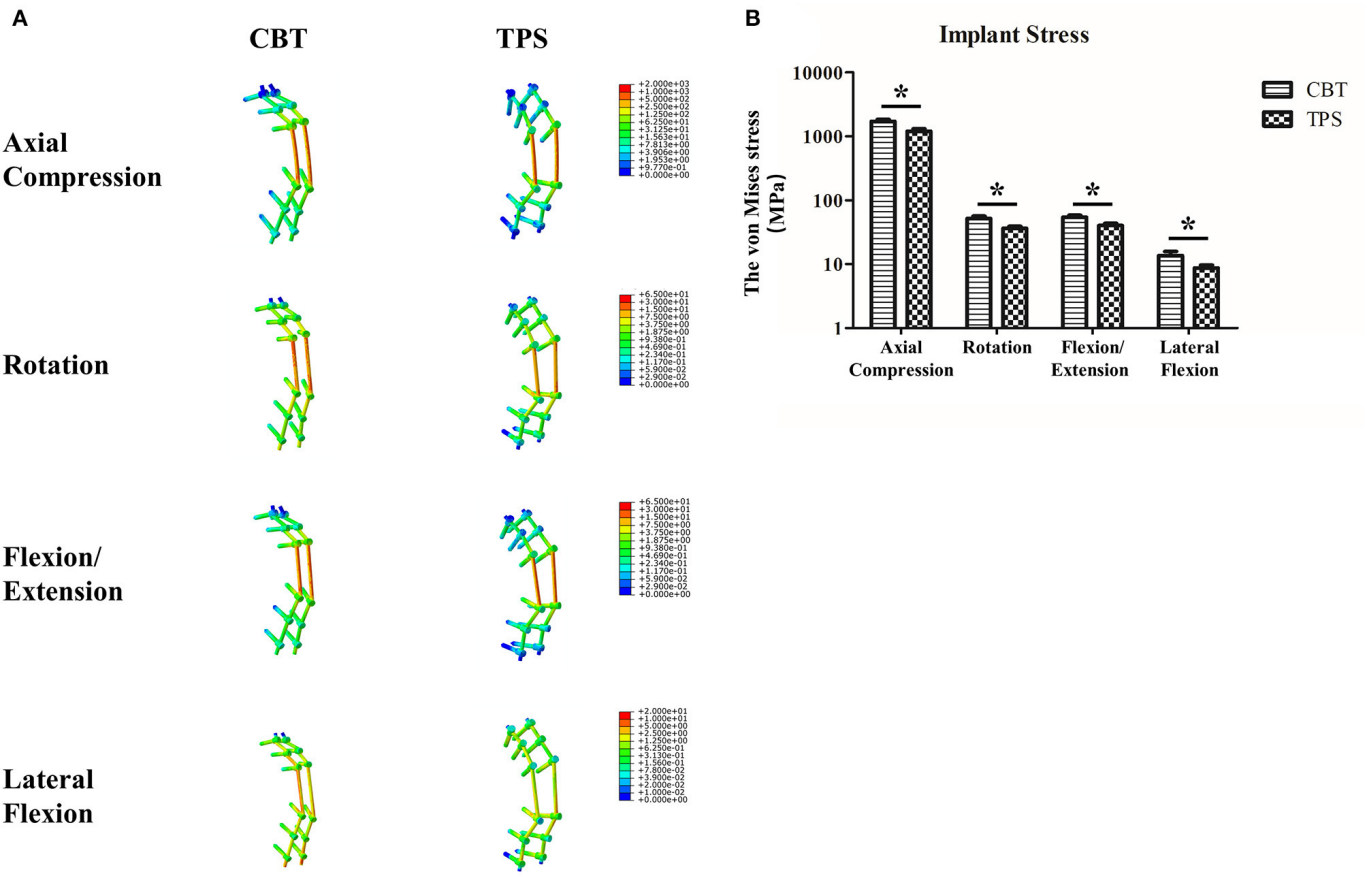
The von Mises stress values and the stress distributions of the implants under axial compression, rotation, flexion/extension, and lateral flexion loads **(A)**. The maximum von Mises stresses of the implants under different loading conditions **(B)**. *p < 0.05.

The maximum von Mises stress values and the distributions of stress on the pedicle screws under different conditions are shown in Figure 3. The von Mises stress was concentrated on the longitudinal rods at the fracture level. Moreover, the bone-screw joints and screw-rod joints had high stress concentrations. The maximum von Mises stress values were 1,714.4 ± 129.8 MPa and 1,208.7 ± 107.3 MPa (*p* < 0.001), which occurred in the CBT and TPS groups under compressive loading. The von Mises stresses of the implants were small under the other loading conditions.

## Discussion

AS is a form of chronic immune-mediated arthritis that often leads to spondyloarthritis, peripheral arthritis, enthesopathy, and anterior uveitis (20). The typical feature of AS in the skeletal system is the coexistence of erosive osteopenia and bony overgrowth (3). Over the past few years, remarkable progress has been made in the medical treatment and therapeutic management of AS. However, AS fractures lead to a sharp decline in patients' quality of life and need to be treated by surgery (7–10). The goal of surgery is to restore sagittal alignment, reconstruct compressed vertebrae, relieve neurologic compression, achieve stable fixation, and enhance bone regeneration (8, 9). Pedicle screw fixation through the transpedicular approach has been suggested to be the ideal fixation method to treat thoracolumbar spinal fractures (7, 8). However, pedicle screw pull-out is still one of the most common complications that occur in patients with poor bone quality (7, 10). To prevent this complication, the CBT technique has been used to enhance the screw holding force in the cortical bone pathway (11). Therefore, understanding the biomechanical characteristics of the CBT and TPS techniques is of great significance for clinical treatment.

Cadaveric biomechanical research on AS fractures is difficult to perform due to the limited availability of cadaveric specimens and ethical dilemmas. Therefore, finite element analysis is an ideal method to analyze the biomechanical properties of AS fractures. Muheremu et al. developed a finite element AS model with kyphosis for biomechanical analysis (21). The authors found that a finite element model can serve as a reliable electronic platform for preoperative planning and biomechanical analysis regarding kyphotic AS. Robinson et al. established an AS finite element model and analyzed the biomechanical characteristics of long posterior stabilization for cervicothoracic fractures (22). The results suggested that a long construct enhanced the stabilization of fracture segments, reducing the instrumentation used did not reduce construct stability, and instrumentation reduced the stress in the ossifications, regardless of the length or whether some instrumentation was not used. Matsukawa et al. performed a series of finite element studies on the CBT technique to provide biomechanical reference data for clinical decision making (16). To our knowledge, the biomechanical characteristics of the CBT and traditional transpedicular techniques for pedicle screw insertion in thoracolumbar AS fracture fixation have not been studied by finite element analysis.

In the present study, compared with the traditional transpedicular technique, the CBT technique showed unsatisfactory biomechanical stability. For intact stability, both the CBT and TPS techniques were able to restore the construct stiffness in the lateral bending simulation. These two methods failed to achieve overall spine stability within compression, rotation, flexion/extension forces after fractures. For fracture segment displacement, the TPS technique yielded better local biomechanical stability than did the CBT technique. The local stability between the proximal and distal fracture segments under lateral flexion loads was almost three times less in the TPS group than in the CBT group, which is consistent with the results of previous studies. These unexpected results may be caused by abnormal quality of the cortical and cancellous bone in the presence of AS. In the presence of AS, not only the bone mineral density but also the anatomical structure of bone is abnormal. Abnormal bone conditions decrease the quality of contact between bones and screws. Moreover, the CBT technique is limited by the anatomical distribution of cortical bone, so it is difficult to achieve spinal three-column fixation. The three-column concept was proposed to illustrate the biomechanical characteristics of the load bearing and supporting structures the thoracolumbar spine. The middle column is considered as the critical part to stabilize fractures. The TPS technique could stabilize the anterior and middle columns which provides stronger fixation strength and better local stability.

The CBT technique tends to enhance the screw holding force and prevent instrumentation failure. However, recent studies have shown inconsistent results regarding the advantages of CBT. A cadaveric study showed that there were no significant differences in stability between the CBT and the traditional TPS technique (23). A biomechanical study pointed out that the CBT technique provides adequate stiffness under flexion/extension and axial rotation loading conditions with rescue screws after instrumentation failure (24). Matsukawa et al. found that CBT provided better biomechanical results than did the traditional transpedicular technique in lumbar fixation (16). CBT provided better biomechanical results under cephalocaudal, mediolateral, flexion/extension loading conditions than did the traditional transpedicular technique in lumbar fixation. Inferior biomechanical characteristics were shown under lateral bending and axial rotation loading conditions. Moreover, the authors compared the mechanical strength of spondylotic vertebrae treated with the CBT and the traditional TPS technique (25). Compared with the TPS technique, the CBT technique showed worse fixation strength and stabilization for axial pullout strength, flexion/extension and lateral bending forces, and axial rotation. The authors explained that the solid purchase defects of the cortical bone of the lamina led to unsatisfactory CBT technique results. These inconsistent conclusions limit the future application of the CBT technique in the treatment of AS thoracolumbar fractures.

There were some limitations of our study. First, the finite element models were created based on the basic skeleton-ligament system, and the effects of muscles and other soft tissues were not considered. Second, although many physical properties of AS bone are very different from those of normal bone, we created the AS model by adjusting only the elastic modulus and density of the tissue and did not adjust other physical properties. This method is similar to the methods used in other finite element studies (21, 22). Third, we examined only early post-operative stability without evaluating long-term biomechanical characteristics. Fourth, an extreme unstable three-column thoracolumbar fracture was simulated in present study. However, the current model cannot represent all types of AS fracture. Considering the limitations of our study, the conclusions should be interpreted with caution from a clinical perspective.

In conclusion, the present study revealed the biomechanical characteristics of the CBT and TPS in AS spine fixation. The TPS technique provided better biomechanical strength under axial, rotational, flexion/extension, and lateral flexion loading conditions than did the CBT technique. Compared with CBT, TPS was more effective in maintaining the stability of AS thoracolumbar fractures from a finite element analysis perspective.

## Data Availability Statement

The raw data supporting the conclusions of this article will be made available by the authors, without undue reservation.

## Ethics Statement

This study was approved by the Ethics Committee of Beijing Jishuitan Hospital. All patients provided written informed consent before study commencement.

## Author Contributions

ZT and YZ conceived the study, designed the study protocol, and edited the final draft of the manuscript. ZT, BX, and YZ analyzed the data and wrote the initial draft of the manuscript. YX, KY, ZT, BX, and YZ were involved in implementing trial and data collection. All authors contributed to the article and approved the submitted version.

## Conflict of Interest

The authors declare that the research was conducted in the absence of any commercial or financial relationships that could be construed as a potential conflict of interest.

## Publisher's Note

All claims expressed in this article are solely those of the authors and do not necessarily represent those of their affiliated organizations, or those of the publisher, the editors and the reviewers. Any product that may be evaluated in this article, or claim that may be made by its manufacturer, is not guaranteed or endorsed by the publisher.
